# De la salud colectiva a la medicina “personalizada”: desafíos bioéticos de la evaluación genética preimplantatoria desde la perspectiva norte-sur

**DOI:** 10.18294/sc.2023.4481

**Published:** 2023-09-12

**Authors:** Natacha Salomé Lima, María Alejandra Petino Zappala, Ailin Delvitto, Miguel Adrián Romero, Nahuel Pallitto

**Affiliations:** 1 Doctora en Psicología. Investigadora Asistente, Consejo Nacional de Investigaciones Científicas y Técnicas, con sede en Facultad de Psicología, Universidad de Buenos Aires, Ciudad Autónoma de Buenos Aires, Argentina. lima.natacha.s@gmail.com Universidad de Buenos Aires Facultad de Psicología Universidad de Buenos Aires Ciudad Autónoma de Buenos Aires Argentina lima.natacha.s@gmail.com; Consejo Nacional de Investigaciones Científicas y Técnicas; 2 Doctora en Ciencias Biológicas. Becaria postdoctoral, Consejo Nacional de Investigaciones Científicas y Técnicas, con sede en Facultad de Filosofía y Letras, Universidad de Buenos Aires. Jefa de Trabajos Prácticos, Facultad de Ciencias Exactas y Naturales, Universidad de Buenos Aires, Ciudad Autónoma de Buenos Aires, Argentina. mapz@ege.fcen.uba.ar Consejo Nacional de Investigaciones Científicas y Técnicas Facultad de Ciencias Exactas y Naturales Universidad de Buenos Aires Consejo Nacional de Investigaciones Científicas y Técnicas Ciudad Autónoma de Buenos Aires Argentina mapz@ege.fcen.uba.ar; 3 Licenciada en Ciencias Biológicas. Becaria doctoral, Consejo Nacional de Investigaciones Científicas y Técnicas, con sede en Facultad de Ciencias Exactas y Naturales, Universidad de Buenos Aires, Ciudad Autónoma de Buenos Aires, Argentina. delvitto.ailin@gmail.com Consejo Nacional de Investigaciones Científicas y Técnicas Facultad de Ciencias Exactas y Naturales Universidad de Buenos Aires Consejo Nacional de Investigaciones Científicas y Técnicas Ciudad Autónoma de Buenos Aires Argentina delvitto.ailin@gmail.com; 4 Licenciado en Psicología. Investigador, Facultad de Psicología, Universidad de Buenos Aires. Asesor pedagógico de formación docente, Ministerio de Educación, Ciudad Autónoma de Buenos Aires, Argentina. miguel.romero@bue.edu.ar Universidad de Buenos Aires Facultad de Psicología Universidad de Buenos Aires Ciudad Autónoma de Buenos Aires Argentina miguel.romero@bue.edu.ar; Ministerio de Educación; 5 Doctor en Filosofía. Investigador Asistente, Consejo Nacional de Investigaciones Científicas y Técnicas, con sede en Facultad de Filosofía y Letras, Universidad de Buenos Aires, Ciudad Autónoma de Buenos Aires, Argentina. nahuelpallitto@conicet.gov.ar Consejo Nacional de Investigaciones Científicas y Técnicas Facultad de Filosofía y Letras Universidad de Buenos Aires Consejo Nacional de Investigaciones Científicas y Técnicas Ciudad Autónoma de Buenos Aires Argentina nahuelpallitto@conicet.gov.ar

**Keywords:** Bioética, Medicina de Precisión, Medicina Reproductiva, Pruebas Genéticas, Reproducción, Salud Reproductiva, Salud Colectiva

## Abstract

El artículo se interroga por los alcances y los límites del paradigma de la medicina de precisión y su relación con el enfoque de la salud colectiva. Para ello, se toma la evaluación genética preimplantatoria o PGT (preimplantation genetic testing) dado que constituye un ejemplo paradigmático de tecnologías que apuntan a la “individualización” de los procesos de salud. En esta dirección, se revisan las características y los fundamentos científico-normativos acerca de las tecnologías PGT en Argentina, y el camino que queda por recorrer para su análisis bioético. De manera más específica, se visibilizan algunas de las condiciones de posibilidad para su implementación desde la perspectiva norte-sur. Como síntesis del análisis, proponemos tres ejes o nudos problemáticos relacionados con los sesgos en la producción de conocimiento, los valores e intereses subyacentes a sus usos y los presupuestos epistemológicos que operan en la base de estas tecnologías. A lo largo de este trabajo, presentamos estos dilemas y sugerimos algunas recomendaciones para ser tenidas en cuenta en futuras investigaciones.

## INTRODUCCIÓN

El paradigma de la medicina de precisión, entendida como una “forma de acercamiento a la prevención, diagnóstico y tratamiento de enfermedades que considera la variabilidad individual”^1^ propone enfoques “personalizados” que se basan en un conocimiento genético específico, para intentar responder a problemas de salud complejos. Una de las primeras preguntas que nos hacemos es de qué manera este paradigma, cuyo modelo apunta a la individualización, responde o se integra a las necesidades de la salud colectiva. 

Para analizar este interrogante, decidimos trabajar con un conjunto de tecnologías -las evaluaciones genéticas preimplantatorias- en el contexto de la reproducción asistida. Dichas tecnologías constituyen una de las formas actuales del paradigma de la medicina de precisión que ya cuentan con cierta legitimación y con prácticas institucionalizadas. De allí que su elección resulte significativa para evaluar las reflexiones y discusiones de la bioética ante el auge de la medicina de precisión.

En términos generales, la evaluación genética preimplantatoria -*preimplantation genetic testing* (PGT)- consiste en conocer la estructura o composición genética de un embrión antes de que sea transferido al útero, otorgando a quienes atraviesan un proceso de fertilización asistida la posibilidad de seleccionar embriones que sean considerados *deseables*, de acuerdo a algún parámetro genético vinculado con la salud-enfermedad. Como mencionaremos más adelante, existen distintas formas de PGT que dependen del tipo y la cantidad de variantes genéticas que se analicen. Pese a su actual utilización en la mayoría de los centros de reproducción locales, en Argentina no abundan los estudios bioéticos sobre estas tecnologías, y menos aún se hallan reflexiones actuales que las encuadren dentro de un marco de salud colectiva y con una perspectiva latinoamericana. Algunos antecedentes de este tipo fueron realizados en otros campos prácticos, como los vinculados a los delitos sexuales^2^ y la selección de trabajadores en función de sus perfiles genéticos^3^, en tanto aquellos directamente vinculados con las tecnologías reproductivas se realizaron en su mayoría antes de las reglamentaciones vigentes bajo condiciones contextuales diferentes^4^^,^^5^^,^^6^. Los estudios actuales se han focalizado en las percepciones respecto de los aspectos éticos del PGT en Argentina^7^ y sus resultados iluminan la importancia de estudiar los aspectos éticos de la práctica reproductiva en su contexto específico de ocurrencia. 

El análisis efectuado en este artículo parte de revisar las características y los fundamentos científico-normativos acerca de las tecnologías PGT en Argentina, y el camino que queda por recorrer para su análisis bioético. En particular, nos interesa visibilizar algunas de las condiciones locales que interpelan nuestros modos de pensar el desarrollo científico tecnológico y la innovación en esta área. Más precisamente, buscamos mostrar, a partir de la problematización de las categorías que intervienen o participan en los debates bioéticos sobre evaluación genética preimplantatoria, las líneas de indagación futuras. A su vez, el trabajo busca delimitar aquellas categorías o discusiones que se originan en otras latitudes -es decir, que provienen de “otros mundos”- y que no necesariamente tienen relevancia en nuestros territorios. Es por ello que el eje global-local o norte-sur será una categoría que transversalmente acompañará los análisis propuestos para mantener una actitud de sospecha respecto de la tecnología que importamos. Así, el análisis bioético propuesto busca visibilizar cómo se construye la evidencia científica, de dónde proviene, cómo se evalúa a nivel local (qué actores participan de la evaluación y qué roles cumplen) y qué efectos supone su traslación clínica. 

En las siguientes secciones se desarrolla una dimensión o un aspecto de las tecnologías de evaluación genética preimplantatorias con significancia para un análisis bioético desde el sur global, culminando cada sección con recomendaciones en forma de sugerencias, orientaciones o alertas para los trabajos bioéticos futuros. En el apartado final se ofrecen una serie de conclusiones y una lectura general de lo desarrollado en las distintas secciones. Esperamos que este análisis muestre los marcos de inteligibilidad de los fenómenos que analizamos y esboce algunas líneas para continuar el trabajo bioético latinoamericano desde una perspectiva de salud colectiva.

## EL CONTEXTO MÉDICO-LEGAL DE LA REPRODUCCIÓN ASISTIDA EN ARGENTINA

En los últimos años, el ámbito de la medicina reproductiva en Argentina participó de una transformación legislativa que marcó un cambio en las prácticas y procedimientos asistenciales, en al menos dos hitos normativos: la sanción en 2013 de la Ley 26862 de *acceso integral* a la cobertura de los tratamientos de reproducción asistida y, posteriormente, la reforma legislativa en 2015 del Código Civil y Comercial, que incluyó la filiación por tecnologías reproductivas^8^. 

Junto a un contexto normativo habilitante para el desarrollo tecnológico y la ampliación de los derechos sexuales y reproductivos para muchas personas que antes no podían acceder a un tratamiento de estas características, comenzó a diversificarse la oferta de centros de fertilidad -actualmente ubicados en su mayoría en la región metropolitana de Buenos Aires (AMBA), pero también en centros urbanos de las provincias de Córdoba, Santa Fe y Tucumán- y de tecnología asociada a estos procedimientos. Como ya mencionamos, una de esas tecnologías, las PGT, son el objeto de este análisis. 

El marco regulatorio nacional brindó un paraguas para el desarrollo, crecimiento, y consolidación de estas tecnologías en nuestro país; sin embargo, la falta de una regulación específica sobre temas sensibles como el destino de los embriones criopreservados, los plazos para la criopreservación, o la inclusión de los test genéticos preimplantatorios dentro de la cobertura que regula el Artículo 8 de la Ley 26862 genera controversias y paradojas en el acceso. La falta de una ley especial que regule la protección del embrión fecundado in vitro ha llevado a la judicialización de los casos en las distintas cortes de nuestro país exponiendo las dificultades que, en el contexto argentino, aparecen a la hora de decidir qué hacer con los embriones criopreservados^9^. 

Las controversias legislativas han llevado a una abundante producción de textos normativos vinculados con los dilemas que presenta el uso de estas tecnologías en nuestro país, identificando, en muchos casos, las cuestiones legales con las propiamente éticas. Si bien las discusiones legales son indicadores de cómo social y culturalmente nos posicionamos ante los dilemas del inicio de la vida, la bioética debe ser capaz de ofrecer marcos conceptuales que no queden subsumidos en la perspectiva legal. 

Recomendación 1: *Preservar la autonomía de la reflexión bioética con respecto a la dimensión legal; aspecto que, en Argentina, no se encuentra correctamente diferenciado.*

## CARACTERÍSTICAS Y PROCEDENCIA DE LOS DATOS CLÍNICOS SOBRE PGT

Otro de los problemas que encontramos para conducir análisis de tipo bioético en América Latina es la escasez de datos sobre la realidad clínica de estos procedimientos (cantidad de casos, indicaciones, y usos de estas tecnologías). Los reportes de la RedLara muestran que, entre 2014 y 2018, el uso del PGT se duplicó de un 14% a un 28% en la región^10^. Es interesante que la explicación que se da de este fenómeno incluye el aumento del PGT en casos de donantes de óvulos jóvenes (un 19,3% de los casos de PGT registrados), algo que les resulta “difícil de comprender”, a lo que añaden que cada vez más “mujeres y hombres parecen no estar preparados para enfrentar cualquier forma de incertidumbre”^10^.

Al considerar el caso argentino, dentro de la producción local, una de las fuentes de consulta de referencia es el registro que produce la Sociedad Argentina de Medicina Reproductiva, que nuclea a los centros de fertilidad del país. El registro se conforma a partir de la información que estas instituciones reportan *voluntariamente* sobre algunos procedimientos (cantidad de tratamientos de donación de óvulos y esperma, cantidad de embriones transferidos, entre otros) pero no incluye información sobre procedimientos de evaluación genética preimplantatoria. Los datos que se publican, en general, son producidos por los mismos grupos de profesionales que ejercen su labor en el área de la reproducción asistida, sobre todo pertenecientes al sector privado y nucleados en colectivos y/o sociedades científicas^11^^,^^12^^,^^13^. Una parte importante de la información también está presente en los sitios web de los centros de fertilidad, destinada a personas con o sin dificultades reproductivas, que son potenciales pacientes o usuarios de tecnologías reproductivas. 

Recomendación 2: *Contemplar que los datos científicos asociados a las tecnologías reproductivas son escasos, provienen de las mismas instituciones que ofrecen servicios en el mercado de la reproducción asistida y son de origen privado. Esto resulta importante en la medida en que la poca información científica disponible se encuentra atravesada por intereses comerciales, que no pueden soslayarse*. 

## LA CUESTIÓN TERMINOLÓGICA: CAMBIOS EN LA FORMA DE DECIR Y EFECTOS EN EL HACER

Si bien las tecnologías reproductivas están disponibles en Argentina hace más de 30 años, su origen, consolidación y desarrollo proviene de los países centrales. De acuerdo al estudio de Okhovati *et al*.^14^, según la cantidad de artículos científicos publicados en la base de datos Medline entre 1998 y 2014, los países que lideraban la investigación en tecnologías reproductivas en ese período eran EEUU (16.453 publicaciones), Reino Unido (5.427 publicaciones), Japón (4.805), China (4.660) y Francia (3.795). Esto muestra que la investigación mundial sobre tecnologías reproductivas se distribuye geográficamente y se concentra entre los países más enriquecidos del mundo^14^. Entender cómo se traducen esas investigaciones en el contexto local, y cómo se piensa la traslación a la clínica, supone hacer un trabajo de interpretación e incluso de dar sentido a la recepción de tecnologías “extranjeras” en el contexto local. Esta traducción no es solo terminológica; como se verá a continuación la forma de nombrar a las diferentes tecnologías tiene múltiples efectos tanto a nivel de las prácticas clínicas como de los entornos normativos que se encargan de regularlas. Es en las formas de decir donde pueden ser analizados los sentidos y los valores en juego. Como señala Haraway^15^ “los términos se mueven unos dentro de otros, son sedimentaciones cambiantes de lo más importante del mundo: la relacionalidad. Es extraño, la relacionalidad encarnada es la profilaxis tanto para el relativismo como para la trascendencia. Nada viene sin su mundo”^15^. 

En este sentido, es conveniente comenzar por mostrar cómo ha cambiado la forma de comprender las tecnologías preimplantatorias a partir de un ajuste en la nominación y alcance de sus propósitos. Los términos de *diagnóstico* y *screening genético preimplatatorio* (PGS y PGD respectivamente) fueron reemplazados por *evaluación genética preimplantatoria* (PGT) luego de una revisión de la terminología utilizada para la atención en salud reproductiva, publicada en *The international glossary on infertility and fertility care*^16^. Uno de los cambios más importantes fue quitar la palabra *diagnóstico* para enfatizar que se trata de una *evaluación* que, en algunos casos, puede ser diagnóstica para condiciones genéticas específicas, pero que en otros casos el resultado indica una probabilidad de presentar un riesgo asociado a múltiples variables. De esta manera, no se trata solo de la posibilidad *diagnóstica* entendida en sentido tradicional. Esto interroga, a su vez, el alcance de la noción de *salud* y de los criterios de *salud-enfermedad* como principios rectores en la aplicación de estas tecnologías y se abre a otros sentidos, como la selección de embriones por sexo y parámetros “no médicos”. Se observa cómo el alcance de las tecnologías reproductivas no encuentra su límite en la *reproducción*, sino que interpela los sentidos asociados a la salud, a la enfermedad, el riesgo, la discapacidad, el bienestar, la calidad de vida, entre otros (Cuadro 1).

**Cuadro 1:** Cambios en la terminología y nuevos modelos de la evaluación genética preimplantatoria.

Terminología en desuso	Nueva terminología
PGD = Preimplantation genetic diagnosis (Diagnóstico genético de preimplantatorio)	PGT = Preimplantation genetic testing (Evaluación genética preimplantatoria)
PGS = Preimplantation genetic screening (cribado genético implantatorio)	PGT-a = Preimplantation genetic testing for aneuploidy (Evaluación genética preimplantatoria para aneploidías).
Tipos de evaluación genética preimplantatoria, según indicación médica	
PGT-SR = Preimplantation genetic testing for structural rearrangements.(Evaluación genética preimplantatoria para rearreglos estructurales)	
niPGT-a = Noninvasive preimplantation genetic testing for aneuploidies. (Evaluación genética preimplantatoria no invasiva para aneuploidías)	
PGT-P = Preimplantation genetic testing for polygenic risk. (Evaluación genética preimplantatoria para riesgo poligénico)	

Fuente: Elaboración propia con base en The International Glossary on Infertility and Fertility Care^16^.

Estos cambios en la forma de decir tienen efectos concretos en las prácticas, por ejemplo, al no tratarse ya de un “diagnóstico” es preciso conocer cómo se realizará la evaluación del riesgo, y qué supone en cada caso presentar un riesgo genético. Esta situación resulta especialmente problemática al haberse expandido los PGT a las condiciones poligénicas, aquellas patologías multifactoriales, en muchos casos de comienzo adulto (como diabetes, hipercolesterolemia o trastornos de salud mental) en las que resulta debatible el peso relativo de los factores genéticos y ambientales, así como la posibilidad concreta de encontrar variantes genéticas causales^17^^,^^18^. Este tipo de PGT se asocia a una creciente preocupación por la optimización de la salud y extiende la vigilancia genética a casos en que no existen antecedentes familiares de enfermedad^19^. Tampoco son ofrecidos exclusivamente a personas que atraviesan una situación de infertilidad; ya no solo son presentados como una vía para tener hijos, sino hijos *más saludables*^20^. Como veremos más adelante, en los casos de PGT, el riesgo se encuentra asociado muchas veces a una responsabilidad. En los casos más extremos, esta responsabilidad genética de los futuros progenitores alcanza a cualquier característica que se asocie a “la mejor vida posible”, que no solo haría al uso de la tecnología de PGT aceptable, sino que lo transformaría en un imperativo moral, incluso si la consecuencia de estas acciones aumenta las desigualdades sociales^19^. 

Recomendación 3: *Reconocer los efectos del lenguaje involucrados en el discurso científico y clínico vinculado con las tecnologías reproductivas, que se relacionan con los corrimientos que se están evidenciado en el contexto del diagnóstico de enfermedades al establecimiento de un riesgo, y de las condiciones de clara base genética hacia la optimización general de la salud, con el borramiento de los factores ambientales, que excluyen especialmente aquellos de tipo social y relacionados con los modos de vida. En este sentido, podemos enmarcar el crecimiento de los PGT en la lógica del “saludismo”*^21^*y la responsabilización individual por la salud que, como veremos en las próximas secciones, se han hecho varias críticas en este sentido desde el marco de la salud colectiva.*

## ANÁLISIS DEL PGT DESDE LA MATRIZ RIESGO-SUSCEPTIBILIDAD

Como señalamos antes, pasar del “diagnóstico” al análisis del “riesgo” supone poder dar cuenta cómo se construye y se explica esta noción en este ámbito. Se ha mostrado que, en el contexto de las pruebas genéticas, aparecen dificultades a la hora de interpretar los resultados y, por ende, cobra relevancia la forma de comunicar y transmitir información en términos de “riesgo”. La problematización del concepto de *riesgo* usualmente se enfoca en los sesgos cognitivos que dificultan interpretar porcentajes o proporciones^22^. En el caso de las estimaciones de riesgo genético, las reacciones negativas y la sobreestimación de los riesgos (o su interpretación en términos deterministas) son habituales^23^^,^^24^. Por esos motivos se plantea la importancia de que los estimadores de riesgo sean comunicados de forma que su magnitud no sea sobreestimada por el paciente, no se minimicen los posibles efectos psicológicos nocivos de recibir esta información, y se garantice así la toma de decisiones autónomas e informadas. 

En ese sentido, uno de los aspectos más abordados en la literatura sobre la comunicación del riesgo en el ámbito de la salud es el uso de estimadores absolutos o relativos. Para ilustrar la diferencia, se puede recurrir al ejemplo del “*contraceptive pill scare*” ocurrido en 1995, cuando el Comité de Seguridad de Medicamentos de Gran Bretaña anunció que el uso de pastillas anticonceptivas duplicaba el riesgo de trombosis con respecto a quienes no las consumían (aumento en el riesgo relativo). Sin embargo, en términos absolutos esto significaba un aumento de solo 1 en 7.000 personas; el incidente causó que decayera el consumo de pastillas anticonceptivas y aumentasen los embarazos no deseados^25^. Dado que los riesgos relativizados siempre representan un número mayor que impacta más en el público e introduce un sesgo a favor de la intervención, se recomienda comunicar siempre en términos absolutos^26^.

Sin embargo, estas recomendaciones abordan la forma de comunicar el riesgo, no así las problemáticas asociadas al concepto de riesgo y a la configuración de sujetos “en riesgo”. En primer lugar, Bianchi^27^ argumenta que la configuración de sujetos en riesgo o susceptibles implica un énfasis en la intervención para dirigir a esos sujetos a un futuro “más deseable”, y que las tecnologías genéticas (entre otras) constituyen un “salto cualitativo” en la capacidad de hacer calculable este futuro^27^. En el caso que nos ocupa, como en tantos otros, involucran una estimación de riesgos e intervenciones en ausencia de patologías manifiestas. En el “riesgo” y la “susceptibilidad” entran en tensión los individuos y las poblaciones a las que se quiere regular. Por un lado, podríamos resaltar las críticas a la aplicación clínica del riesgo, que involucra esta tensión entre su cálculo como estimador de una propiedad poblacional y su extensión a los individuos^28^^,^^29^. Almeida Filho *et al*.^30^ argumentan en contra de tal extensión y de otros supuestos y simplificaciones del paradigma actualmente dominante en la epidemiología^30^ y que también encontramos en los supuestos de la medicina de precisión. Por otro lado, problematizan la reificación del riesgo y su uso culpabilizante, de reforzamiento de “contenidos morales y conservadores”, su rol en la creación de nuevos mercados en la prevención de múltiples riesgos y en la expansión de la vigilancia médica con el objetivo de extender la longevidad lo máximo posible^30^. Asimismo, junto con otros autores, critican desde una perspectiva norte-sur las categorías utilizadas, los supuestos epistemológicos de la epidemiología clásica, y cómo las concepciones de salud y enfermedad han sido “importadas” o incluso impuestas de forma de ocultar el rol de las relaciones de poder en los procesos de salud, enfermedad y atención, y de minimizar los orígenes y las consecuencias políticas de la utilización de tales conceptos^30^^,^^31^^,^^32^^,^^33^^,^^34^. 

Recomendación 4: *Mantener una mirada crítica sobre las categorías usualmente empleadas en la epidemiología y en la medicina de precisión. La bioética latinoamericana no debería únicamente pensar y discutir cuestiones vinculadas con la accesibilidad, la implementación o el uso de estas tecnologías, ni debería solamente reflexionar acerca de la comunicación de resultados a los pacientes, sino que debe ser capaz también de problematizar los sentidos y expectativas que conllevan las diversas formas de conceptualizar la información genética, para situar sus implicancias y alternativas.*

## DILEMAS BIOÉTICOS “CENTRALES” SOBRE LA EVALUACIÓN GENÉTICA PREIMPLANTATORIA

En esta sección se describen los dilemas bioéticos que pueden ser encontrados en las guías de buenas prácticas y en los documentos normativos de los *países centrales* para poder establecer un contrapunto cuando en el apartado siguiente se analice la traducción o los problemas *periféricos* al uso y aplicación de estas tecnologías.

El análisis de los documentos normativos de países europeos establece que (con excepción del PGT-P) las variantes de PGT suelen ser indicadas para aquellas personas que presentan un *riesgo genético*^35^^,^^36^^,^^37^ y que desean evitar transmitir un fenotipo patológico a su descendencia, o parejas con un riesgo alto de producir embriones con anomalías genéticas, entre ellas personas con edad materna avanzada, que hayan atravesado abortos espontáneos recurrentes y repetidas fecundaciones in vitro fallidas.

En los últimos años, las distintas tecnologías PGT han generado diversos debates y controversias, destacándose los interrogantes acerca de posibles efectos y riesgos para la salud humana. Entre los argumentos a favor de los PGT, se ha hecho hincapié en el derecho a la *autonomía reproductiva* de los consultantes. La autonomía reproductiva es parte de los reconocidos derechos reproductivos y se refiere a la capacidad de los individuos a tomar decisiones informadas y autónomas sobre su propia reproducción. Además, se incluyen como posibles beneficios la capacidad de prevenir la transmisión de desórdenes genéticos, la reducción de interrupciones de embarazos basados en pruebas prenatales, como también los posibles beneficios sociales de reducir la carga global de enfermedades crónicas graves^38^. 

Por otro lado, argumentos en contra de estas tecnologías cuestionan la validez de los beneficios de este procedimiento debido a su carácter especulativo, en tanto hay una posibilidad de error en los resultados o estimaciones de riesgo, una inhabilidad de predecir el desarrollo o proceso de la enfermedad a largo plazo y posibles riesgos aún desconocidos de los procedimientos^39^^,^^40^^,^^41^. También surge la preocupación de que el uso de esta tecnología para la *selección de embriones* contribuya a la devaluación de ciertas vidas^42^; sobre todo, de aquellos sujetos afectados por las enfermedades o que son portadores de determinados marcadores genéticos. Así, se plantea que se refuerzan nociones problemáticas acerca del rol de la genética como causa de la enfermedad, lo que deviene en una tensión entre la *autonomía reproductiva* y la *responsabilidad genética*, ya mencionada en la sección anterior. Estos debates son de larga data. En 1991, Lippman señaló que las tecnologías de exámenes prenatales, si bien se presentan como herramientas para la toma de decisiones informadas acerca de la salud de los hijos, pueden también exacerbar la responsabilidad individual sobre la carga de las distintas enfermedades, reforzando las desigualdades sociales y económicas ya existentes. Esta misma crítica es esgrimida por autores latinoamericanos del campo de la salud colectiva^30^^,^^31^^,^^43^. Como también adelantamos en la sección anterior, la tendencia a atribuir bases genéticas a condiciones multifactoriales alimenta la expansión de los PGT y puede enmarcar el proceso de “genetización” de la sociedad^39^.

En su mayoría, el análisis de las racionalidades de algunos comités de bioética del norte global^35^ reconocen éticamente aceptable la práctica de PGT para aquellas *enfermedades graves* que no cuentan con tratamientos efectivos hasta el momento^38^. En particular, se recomienda como fundamental la participación de un “consejero genético” -tal como fue definido por la OMS en 1969^44^- con experiencia en estos casos durante la consulta para una evaluación adecuada de los beneficios y riesgos potenciales, para así tomar decisiones informadas y éticamente responsables. El caso del PGT-P presenta particularidades que lo hacen más controversial, ya que en su mayoría se informan riesgos para patologías tratables, la extrapolación de los riesgos poligénicos de una población a otra es discutible, así como la falta de factores ambientales en la estimación. Por estos motivos, sumados a la presión que se ejerce sobre los progenitores, algunos autores argumentan que las dificultades para garantizar la autonomía procreativa y la toma de decisiones informadas se acrecientan en los PGT-P^45^^,^^46^, y que al considerar este tipo de pruebas no pueden aplicarse los mismos criterios que para otros PGT, pese a lo cual no hay aún recomendaciones específicas^35^. Aun así, este tipo de testeos están siendo ofrecidos por clínicas en distintos países, incluyendo algunos de América Latina.

Recomendación 5: *Alcanzar reflexividad crítica sobre las propias categorías del campo de la bioética. Debemos ser capaces de problematizar la procedencia de los conceptos y debates provenientes del campo de la bioética para pensar cómo se aplican en nuestros contextos. Enfatizar, por ejemplo, en la autonomía reproductiva como procedimiento formal predominante de la ética invisibiliza las condiciones materiales de las comunidades de nuestros territorios que no siempre están satisfechas para alcanzar dicha autonomía. Este último aspecto nos conduce a la siguiente sección.*

## SOSPECHA BIOÉTICA: NADA VIENE SIN SU MUNDO

Aunque pocas veces se explicite, toda discusión y argumentación bioética presenta presupuestos ontológicos. Lo que se afirma, discute o problematiza tiene siempre un punto de partida ineludible que consiste en la visión del mundo del argumentante; mundo que depende, a su vez, de la historia de ese pueblo, de su situación geográfica, de sus instituciones, de sus luchas, de su lenguaje. Antes de toda posición bioética debemos ya estar en un mundo concreto y cotidiano con sus entidades, sus sentidos, sus símbolos y sus objetos. Mundo es, entonces, “la categoría de partida de toda otra categoría. Es la vida de todos los días como presupuesto (lo puesto debajo en el tiempo: lo pre-sub-puesto)”^47^. Es lo dado, lo que permanece oculto pero opera como fundamento práctico de nuestras acciones y relaciones. 

La bioética no es una excepción y por ello resulta menester reconocer que sus discusiones e intervenciones emergen de y se fundamentan en cierto mundo. Este reconocimiento, el que todo saber, reflexión o argumento supone un mundo concreto, nos obliga, por lo tanto, a dar un paso atrás. Ya no se trata únicamente de evaluar una tecnología como el PGT desde una perspectiva bioética, sino que ahora resulta necesario incorporar una mirada crítica a los marcos conceptuales comúnmente utilizados. De este modo, podremos dialécticamente alcanzar una forma de evaluación bioética de la tecnología más profunda y significativa, porque estaremos en condiciones de realizar un doble juicio: por un lado, aquel que analiza la tecnología desde las categorías que ofrece el mundo dado e incuestionado; por otro lado, aquel otro juicio que, dando un paso atrás, brinda una *mirada crítica* a los presupuestos en los que se fundan las categorías de la bioética empleadas. 

Recomendación 6: *Revisar los presupuestos ontológicos en los que se fundamentan las categorías y discusiones bioéticas. Siguiendo lo analizado en el eje anterior, la orientación que aquí ofrecemos a la bioética latinoamericana consiste en no perder de vista que las categorías empleadas no son necesariamente extrapolables a otros mundos con otras realidades, como puede ser el caso de los países del sur global.*

## MODOS DE AFIRMAR Y REPRODUCIR LA VIDA

Siguiendo el hilo de los párrafos precedentes, si hablamos de formas diferentes de ser-en-el-mundo, estamos obligados como pensadoras y pensadores de América Latina a iniciar nuestra reflexión desde la ontología presupuesta. La región tiene una larga historia de colonialismo y de imposiciones: formas de vida, hábitos, costumbres, valores, patrones de conducta e instituciones; mundos ajenos, en definitiva. Pero la realidad latinoamericana, el mundo latinoamericano, no es ni será nunca el mundo europeo o estadounidense. Existe un fondo simbólico, mítico, histórico y psicológico que permanece y permanecerá siempre por fuera de esa totalidad o universalidad pretendida, manteniéndose como lo Otro, como la alteridad que no se deja totalizar. Por este motivo, por la plurivocidad de mundos que coexisten actualmente en nuestro continente y en el sur global, debemos ser cautos con los análisis de la bioética y preguntarnos qué particularidades de nuestro mundo latinoamericano resulta necesario tener en cuenta cuando se analiza una tecnología reproductiva como el PGT. O, formulado como interrogantes, ¿qué relevancia presenta la distinción norte global y sur global al momento de pensar desde la bioética la adecuación o deseabilidad de una tecnología como el PGT? ¿Desde qué mundos emergen o deberían emerger los juicios bioéticos vinculados con las tecnologías reproductivas?

En general, cuando la bioética reflexiona sobre una tecnología, lo hace aceptando y tomando como punto de partida un mundo gobernado por una *racionalidad tecnocientífica*. Esto implica considerar como un *a priori* de sentido que las necesidades y problemas de las comunidades encuentran siempre salida y soluciones en los saberes científicos y los desarrollos tecnológicos^48^. En algunos casos, como un “determinismo tecnológico”, cuando la tecnología es tratada como una variable independiente que genera “efectos” sobre los procesos sociales, o bien como un “determinismo social” cuando se la piensa como una variable dependiente donde las decisiones de los grupos sociales determinan el cambio tecnológico^49^. En particular, aquellos saberes, tecnologías y formas de pensar que corresponden a los países centrales, a los que según una mirada lineal y universalista del desarrollo tecnológico y del conocimiento, todas las culturas deberían arribar^50^. 

Resulta así natural que, si pensamos en modos de afirmar y reproducir la vida humana, la respuesta sea un *dispositivo* o un *procedimiento tecnológico* basado en la ciencia. Se asume incluso que estos saberes y tecnologías son de aplicación universal, por lo que no hay ninguna diferencia si se trata de conocimientos y herramientas desarrolladas en otros países del globo: si funciona para un ser humano europeo, funciona también para un ser humano latinoamericano. Específicamente, al referirnos al caso de los testeos genéticos, lo que se ha problematizado tradicionalmente es la falta de datos de poblaciones de países no centrales, incluso se argumenta que los mayores desafíos éticos y científicos que rodean la implementación clínica de la medicina de precisión es la falta de diversidad en las bases de datos genómicos^51^, que harían a las predicciones de riesgo menos fidedignas, pero no se problematizan las lógicas que la tecnología misma impone. Según esta forma de concebir el desarrollo científico y tecnológico, lo que en todo caso habrá que hacer es analizar los modos adecuados de implementar y regular la tecnología. 

No obstante, como hemos sugerido anteriormente, ni los conocimientos ni las tecnologías derivadas son en ausencia de un mundo que les otorgue sentido. Como consecuencia, la bioética latinoamericana debería interrogarse acerca de la aceptabilidad del conocimiento supuesto y de la tecnología desarrollada, porque bien podría suceder que sean mediaciones colonialistas, aunque se presenten como soluciones locales, incluso como medios para “lograr soberanía” o revertir inequidades de larga data entre el norte y el sur global. Así como puede esgrimirse la crítica al universalismo tecnológico, también puede hacerse al “universalismo bioético” que, al minimizar la importancia del contexto, remueve la necesidad de reflexionar sobre la “importación” acrítica de los países centrales a los periféricos^52^. Ante esta tendencia creemos que, para verdaderamente atender a las inequidades que afectan a las comunidades latinoamericanas, la solución no puede ser la aplicación acrítica de tecnologías, sino que debe implicar la consideración de las tecnologías en sí mismas en el contexto particular de nuestras sociedades. ¿Es compatible la tecnología con el ser-en-el-mundo de las comunidades latinoamericanas? ¿Se incorpora naturalmente en los proyectos de vida de la población? ¿Es compatible con las diversas concepciones sobre la reproducción de los habitantes de nuestros territorios? Cuando estas preguntas están ausentes, la bioética asume la vía de discutir e intervenir desde la positividad de un mundo ya presupuesto; positividad que impide ver que una cultura que no dispone de la manipulación embrionaria como posibilidad óntica de la reproducción humana no puede juzgar aspectos éticos que se deriven de la aplicación tecnológica de un dispositivo como el PGT. Tampoco puede hacerlo una cultura que no tiene garantizados aspectos materiales mínimos que posibilitan cierta producción y reproducción de su propia vida y descendencia, aunque aquí las razones sean diferentes. En uno u otro caso, las posibilidades quedan por fuera del horizonte existencial de esas comunidades. 

Recomendación 7: *Repensar cuáles son los fundamentos éticos que sustentan nuestra comunidad de vida latinoamericana y nuestras concepciones de la reproducción, para luego ser capaces de evaluar cuáles son las mediaciones adecuadas que nos conducen a cumplir con nuestros proyectos y finalidades reproductivas. Esto resulta incluso más importante si se considera el asunto desde la perspectiva de la salud colectiva. Explicaremos a continuación por qué.*

## EL PUNTO DE PARTIDA DE LA REFLEXIÓN BIOÉTICA LATINOAMERICANA

El fundamento ético material de una tecnología reproductiva debe ser la afirmación de la vida y la salud reproductiva de una comunidad. En ese sentido, el PGT podría ser considerado una mediación que satisface dicho fundamento. En efecto, lo es hasta cierto punto. Sin embargo, puede que este tipo de tecnologías no sean la mejor mediación para la afirmación de la vida y reproducción de las comunidades de América Latina -el argumento podría incluso extenderse a África y a ciertas regiones de Asia- por los motivos que expusimos anteriormente. La facticidad o cotidianidad de las comunidades de nuestros territorios se encuentra fuertemente marcada por la exclusión y la pobreza. En Argentina, por ejemplo, la pobreza alcanzó el 39,2% en el último semestre del 2022, según datos oficiales^53^. Si el fundamento ético de toda tecnología reproductiva es la afirmación de la vida y su reproducción ¿qué significa este dato para nosotras y nosotros como bioeticistas que discutimos tecnologías reproductivas? ¿Puede obviarse el contexto y analizarse el PGT como si las poblaciones latinoamericanas no existieran de ese modo? Puede obviarse a costa de una inversión problemática: colocar la racionalidad tecnológica por delante de la racionalidad ética. Uno de los potenciales problemas que enfrenta la bioética latinoamericana cuando discute tecnologías reproductivas, desde el punto de vista de la salud colectiva, puede ser precisamente el olvido ontológico que conduce a una inversión entre el fundamento y la mediación fundada. Cuando se analiza el PGT (mediación para la vida), se parte de la tecnología (lo fundado) para discutir sus aspectos éticos (el fundamento), invirtiendo el orden de la reflexión. En vez de interrogar por el sentido de una tecnología que surge en otros mundos, la bioética puede dar por sentada su inclusión e indagar sobre las formas éticas de implementarla. 

¿Significa esto que la bioética del sur global debe rechazar todo tipo de PGT o tecnologías semejantes por haber surgido en el norte global? No, no consideramos que deba ser así. Si no más bien mostrar la importancia de hacer un movimiento complementario, aquel que indaga la ontología presupuesta en la tecnología y la enfrenta con la existencia de las poblaciones latinoamericanas. Solo de esta manera es posible pensar una ética desde el sur global con una perspectiva de salud colectiva, una ética crítica que no esconde bajo la alfombra la facticidad de gran parte de nuestra población, que no tiene ni tendrá acceso a dichas tecnologías. Recuperar el fundamento ético que nos orienta -la afirmación de la vida y su reproducción- nos protege del fetichismo tecnológico y nos abre la posibilidad de buscar las mediaciones tecnológicas compatibles con nuestros propios horizontes ontológicos. Y si una tecnología emerge en otros mundos, recordar dicho fundamento ético nos habilita cierta distancia crítica para juzgar a la tecnología en cuestión desde nuestra propia facticidad. Está bien ofrecer a las personas con condiciones genéticas hereditarias que viven en cierta cotidianidad un medio como el PGT para afirmar su vida y su salud reproductiva. Ahora bien: la bioética latinoamericana no puede partir de un mundo europeo o estadounidense para analizarla y juzgarla. Tampoco debe desentenderse del problema de que gran parte de las comunidades que habitan sus territorios existen en la negatividad; por cuestiones materiales o simbólicas, su vida y su reproducción se encuentran negadas. No podemos ocuparnos de la tecnología sin también ocuparnos de eso. 

Recomendación 8: *Comenzar las reflexiones bioéticas latinoamericanas a partir de un análisis de las inequidades o desigualdades en el cuidado de la salud reproductiva, con sistemas de salud fragmentados que vulnerabilizan aún más a las poblaciones con menos recursos, dentro de un mapa que marca una tendencia a la genetización de la salud de algunos en desmedro de la afirmación de la vida y la reproducción de otros, sostenido muchas veces en discursos biomédicos del complejo médico industrial financiero. En esta perspectiva crítica consiste nuestra última orientación para la bioética latinoamericana cuando trata con tecnologías reproductivas como el PGT.*

## CONCLUSIONES

Como hemos intentado argumentar a lo largo de este trabajo, encontramos tres grandes formas de problematizar la adopción de tecnologías reproductivas como el PGT en el contexto latinoamericano. Una de ellas, que puede tal vez considerarse la más tradicional, cuestiona los sesgos de producción de conocimiento que en este caso se hacen evidentes, por un lado, en la subrepresentación de las comunidades latinoamericanas en las bases de datos utilizadas para el cálculo de riesgos genéticos y, por otro, en las dificultades para generar datos fiables sobre la aplicación de las tecnologías en los países de la región, dado que los únicos reportes existentes son voluntarios^10^^,^^54^. Como hemos mencionado, la escasa evidencia disponible es, en gran medida, construida por el sector privado-empresarial y depende también de la voluntad de los centros de fertilidad de brindar información, y de los distintos agentes estatales para crear registros de control y salvaguarda de la información. Por supuesto, estos sesgos epistémicos delatan múltiples relaciones de poder (sector privado-sector público, grandes centros urbanos-regiones periféricas, norte global-sur global) y, de esta forma, recabar datos que permitan implementar y regular estas tecnologías en las poblaciones latinoamericanas se presenta como deseable o incluso un paso casi “emancipatorio”. 

En segundo lugar, señalamos la necesidad de interrogar los valores que subyacen al avance de tecnologías como el PGT. Por un lado, hemos mencionado la “responsabilidad genética” y las nociones de “beneficencia procreativa” que transforman a tecnologías de este tipo en un imperativo moral, a la vez que configuran un mandato sobre quiénes y cómo pueden reproducirse. La expansión de las tecnologías PGT (en términos de la tendencia a realizar cada vez más estudios preimplantatorios, así como el avance de testeos como el PGT-P para condiciones multifactoriales, tratables y de comienzo adulto) muestra el proceso de invasión de “lo médico” en la vida cotidiana. Entre los diferentes tipos de evaluaciones genéticas, tal vez el caso del PGT-P sea el más evidente; sin embargo, muchas veces tampoco es clara la distinción entre razones médicas o no médicas para solicitar o realizar evaluaciones genéticas preimplantatorias. Otros valores, como el de procrear hijos genéticamente vinculados, entran en juego también en la utilización de estas tecnologías y en su regulación. 

Una tercera forma de problematizar estas tecnologías requiere la operación de reflexionar sobre las tecnologías mismas y sus presupuestos en el contexto latinoamericano; dicha reflexión, menos habitual en la literatura sobre estas temáticas, es un ejercicio que puede plantearse desde la salud colectiva y la bioética latinoamericana a través del análisis crítico de las iniciativas de salud en su contexto social^55^. Esto nos lleva a preguntarnos no ya cómo implementar estas tecnologías, sino ¿es deseable hacerlo?, ¿bajo qué circunstancias?, ¿cuáles son los presupuestos de estas tecnologías y qué lógicas pueden estar imponiendo?, ¿constituyen una adecuada mediación para la afirmación de la vida y reproducción de las comunidades?, o ¿en qué contextos pueden serlo?

Para cada uno de los aspectos señalados y trabajados hemos ofrecido una recomendación que esperamos sirva para futuros trabajos o reflexiones de la bioética latinoamericana. Desde luego, lo abordado en este artículo no agota las posibilidades ni las instancias de lo que puede y debe ser discutido desde el sur global cuando se analiza una tecnología reproductiva. Somos conscientes de que la medicina de precisión se expande rápidamente y que ya ha llamado a la puerta de América Latina. Que esta contribución ayude a que nadie nos diga o imponga una respuesta a dicho llamado.
