# Supplementary material for: De la salud colectiva a la medicina “personalizada”: desafíos bioéticos de la evaluación genética preimplantatoria desde la perspectiva norte-sur
Source: Salud Colect. 2023 Sep 12;19:e4481. [Article in Spanish] doi: 10.18294/sc.2023.4481 (PMC11930333; doi:10.18294/sc.2023.4481)
Supplement: Translation

# Translation: From collective health to “personalized” medicine: bioethical challenges in preimplantation genetic testing from a North-South perspective

**Authors:** Natacha Salomé Lima, María Alejandra Petino Zappala, Ailin Delvitto, Miguel Adrián Romero, Nahuel Pallitto

**Affiliations:** 1 PhD in Psychology. Assistant Researcher, National Scientific and Technical Research Council [Consejo Nacional de Investigaciones Científicas y Técnicas, (CONICET)], Faculty of Psychology, Universidad de Buenos Aires, Buenos Aires, Argentina. lima.natacha.s@gmail.com; 2 PhD in Biological Sciences. Postdoctoral Fellow, National Scientific and Technical Research Council, Faculty of Philosophy and Letters, Universidad de Buenos Aires. Assistant Professor, Faculty of Exact and Natural Sciences, Universidad de Buenos Aires, Buenos Aires, Argentina. mapz@ege.fcen.uba.ar; 3 Undergraduate Degree in Biological Sciences. Doctoral Fellow, National Scientific and Technical Research Council, Faculty of Exact and Natural Sciences, Universidad de Buenos Aires, Buenos Aires, Argentina. delvitto.ailin@gmail.com; 4 Undergraduate degree in Psychology. Researcher, Faculty of Psychology, Universidad de Buenos Aires. Pedagogical Advisor in teacher training, Ministry of Education, Autonomous City of Buenos Aires, Argentina. miguel.romero@bue.edu.ar; 5 PhD in Philosophy. Assistant Researcher, National Scientific and Technical Research Council, Faculty of Philosophy and Letters, Universidad de Buenos Aires, Autonomous City of Buenos Aires, Argentina. nahuelpallitto@conicet.gov.ar

**Keywords:** Bioethics, Precision Medicine, Reproductive Medicine, Genetic Testing, Reproduction, Reproductive Health, Collective Health

## Abstract

This article examines the scope and limitations of the precision medicine paradigm and its relationship with the collective health approach. To that end, it takes preimplantation genetic testing (PGT) as a paradigmatic example of technologies aimed at the “individualization” of health processes. In this regard, we review the characteristics and scientific and regulatory foundations of PGT technologies in Argentina, and discuss the next steps for their bioethical analysis. More specifically, we shed light on some of the conditions for their implementation from a north-south perspective. We propose three themes or problematic aspects as a synthesis of our analysis, related to biases in the production of knowledge, the values and interests underlying its uses, and the underlying epistemological assumptions of these technologies. Throughout the article, we review these dilemmas and suggest some issues that should be taken into account in future research.

## INTRODUCTION

The precision medicine paradigm, defined as an “approach to disease prevention, diagnosis and treatment that acknowledges individual variability”^1^ proposes “personalized” approaches based on specific genetic knowledge to attempt to address complex health issues. One of the first questions that are raised in this study is how this paradigm, whose model aims at individualization, addresses or responds to the needs of collective health. 

To analyze this question, we have decided to examine a set of technologies - namely, Preimplantation Genetic Testing (PGT) technologies - in the context of assisted reproduction. These technologies are one of the current forms of the precision medicine paradigm that have attained a considerable degree of legitimacy through institutionalized practices. Thus, this decision becomes significant to evaluate the reflections and discussions in bioethics in response to the rise of precision medicine.

Generally speaking, PGT involves analyzing the genetic structure or composition of an embryo before its implantation in the uterus, with the aim that those undergoing an assisted fertilization process can have the possibility to select quality embryos, based on a genetic parameter linked to health-disease. As will be discussed later, there are various forms of PGT that depend on the type and number of genetic variants being examined. Despite their current use in most local reproductive centers in Argentina, there are only a few bioethical studies on these technologies in the country, and even fewer current analyses developed within the framework of collective health and from a Latin American perspective. While there have been precedents of this type of studies in other practical fields, such as those related to sexual offenses ^2^ and the selection of workers according to their genetic profiles,^3^ research directly related to reproductive technologies was mainly carried out before the current regulations under different contextual conditions.^4^^,^^5^^,^^6^ Recent studies have focused on perceptions associated with the ethical aspects of PGT in Argentina ^7^ and their findings shed light on the importance of addressing the ethical aspects of the reproductive practices within the specific context in which they occur. 

The analysis conducted in this paper arises from a review of the characteristics and scientific-normative foundations of PGT technologies in Argentina, and the road ahead for their bioethical analysis. Specifically, our aim is to make visible certain local conditions that challenge our ways of addressing technological-scientific development and innovation in this field. Moreover, we intend to outline future research directions through the problematization of the categories relevant to the bioethical debates surrounding PGT. Furthermore, this study seeks to circumscribe those categories or discussions that originate in other regions, i.e, those that come from ‘other worlds’ and may not necessarily be relevant in our territories. This is why the global-local or North-South axis will be a guiding category that will transversely support these analyses to maintain a critical attitude towards the technology that we import. Consequently, the proposed bioethical analysis aims to make visible how scientific evidence is constructed, where it comes from, how it is evaluated at the local level (including actors involved and their roles) as well as the effects that are implied in its clinical translation. 

The following sections will develop a dimension or aspect of PGT technologies that is significant for a bioethical analysis from the global South. Each section concludes with recommendations in the form of suggestions, guidelines or warnings for future bioethical studies. In the final section, we include a series of conclusions and an overview of what has been developed in the previous sections. We hope that this analysis may provide a clear understanding of the phenomena that we are examining in this study, outlining some directions to continue the Latin American bioethical work with a focus on collective health.

## MEDICAL-LEGAL CONTEXT OF ASSISTED REPRODUCTION IN ARGENTINA

In recent years, the field of reproductive medicine in Argentina has undergone a legislative transformation that marked a shift in healthcare practices and procedures, with at least two regulatory milestones: the enactment of Act 26862 in 2013, which granted *comprehensive access* to coverage for assisted reproductive treatments and, later on, the legislative reform in 2015 of the Civil and Commercial Code, which included filiation through reproductive technologies. ^(^^8^

This regulatory context has fostered technological advancements and expanded sexual and reproductive rights for individuals who previously could not access such treatments. Consequently, it has led to a diversification of services offered by fertility centers. Most of these centers are currently located in the metropolitan area of Buenos Aires [AMBA in Spanish], but are also expanding into urban centers in the provinces of Córdoba, Santa Fe and Tucumán, increasing access to these procedures. As mentioned earlier, one of these technologies, Preimplantation Genetic Testing (PGT), is the subject of this analysis. 

The national regulatory framework has supported the development, growth, and consolidation of these technologies in Argentina. However, the lack of specific regulations addressing sensitive issues such as the disposition of cryopreserved embryos, the timeframes for cryopreservation, or the inclusion of PGT within the coverage regulated by Section 8 of Act 26862 have given rise to controversies and paradoxes concerning access. The absence of a special law that regulates the protection of *in vitro*-fertilized embryos has resulted in the judicialization of cases in various Argentine courts, revealing the difficulties that arise in the local context when it becomes necessary to determine the fate of cryopreserved embryos.^9^

These legislative controversies brought about a significant production of regulatory texts ddressing the dilemmas arising from the use of these technologies in Argentina, often conflating legal with ethical issues. While legal discussions serve as indicators of how our society positions itself socially and culturally with regard to questions about the beginning of life, bioethics should offer conceptual frameworks that do not become subordinate to the legal perspective.

Recomnendation 1: *Preserve the autonomy of bioethical reflection concerning the legal dimension, an aspect that is not clearly differentiated in Argentina.*

## CHARACTERISTICS AND ORIGIN OF PGT CLINICAL DATA

Another challenge that we encountered when conducting bioethical analyses in Latin America is the scarcity of data regarding the clinical reality of these procedures (number of cases, indications and uses of these technologies). Reports from the Latin American Network of Assisted Reproduction [RedLara] reveal that, between 2014 and 2018, the use of PGT doubled from 14% to 28% in the region.^10^ Interestingly, the explanation for this phenomenon includes the rise in the use of PGT in cases involving young oocyte donors (which accounts for 19.3% of registered PGT cases), a fact that they can “hardly understand”, to which they add that “women and men increasingly seem to be unprepared to face any form of uncertainty.”^10^

When considering the Argentine case, one of the primary sources of reference in the local production is the registry maintained by the Argentine Society of Reproductive Medicine, which brings together fertility centers in Argentina. This registry contains information *voluntarily* reported by these institutions with regard to specific procedures (such as the number of oocytes and sperm donation treatments, the number of implanted embryos, among others) but does not include data on PGT procedures. The available data that is generally published is generated by the same groups of professionals working in the field of assisted reproduction, mainly those in the private sector who organize themselves into collectives and scientific societies.^11^^,^^12^^,^^13^ A substantial amount of this information can also be found on the fertility center websites, targeting individuals with or without reproductive difficulties, who are potential patients or users of reproductive technologies. 

Recommendation 2: *Take into account that scientific data associated with reproductive technologies are scarce, come from the same institutions that provide services in the assisted reproduction market, and are of private origin. This is important, considering that the limited scientific information available is permeated by commercial interests, a fact that cannot be disregarded*. 

## THE TERMINOLOGICAL ISSUE: CHANGES IN HOW THINGS ARE SAID AND THEIR EFFECTS ON HOW THINGS ARE DONE

Although reproductive technologies have been available in Argentina for over 30 years, their origin, consolidation and development have roots in the central countries. According to the study by Okhovati *et al*.,^14^ and considering the number of scientific articles published on the Medline database between 1998 and 2014, the leading countries in reproductive technology research for that period were the USA (16,453 publications), the UK (5,427 publications), Japan (4,805), China (4,660) and France (3,795). This illustrates that global research on reproductive technologies is geographically unequally distributed and concentrated among the richest countries of the world.^14^ Understanding how these research findings are translated locally and how this translation into clinical practice is conceived requires a work of interpretation and also awareness of how “foreign” technologies are received in the local context. This translation is not just about terminology. As will be explored later, the way these technologies are named has numerous effects, influencing both clinical practices and the regulatory environments that oversee them. It is in the way things are expressed that the meanings and values at stake can be analyzed. According to Haraway,^15^ “[t]he terms pass into each other; they are shifting sedimentations of the one fundamental thing about the world - relationality. Oddly, embedded relationality is the prophylaxis for both relativism and transcendence. Nothing comes without its world.”^15^

In this sense, it is convenient to start by showing how the way we understand preimplantation technologies has changed through an adjustment in terminology and the scope of their purposes. The terms “preimplantation genetic diagnosis and screening” (PGD and PGS respectively) were replaced by “preimplantation genetic testing” (PGT) after a review of the terminology used for reproductive health care, published in *The International Glossary on Infertility and Fertility Care.*^16^ One of the most significant changes was to remove the word “diagnosis” to emphasize that it is *an evaluation* that, in certain cases, may be diagnostic for specific genetic conditions, but in other cases, shows a probability of presenting a risk associated with multiple variables. Therefore, it is not just about a *diagnostic* possibility in the traditional sense. In turn, this questions the scope of the notion of *health* and *health-disease* criteria as leading principles in the application of these technologies, and opens up to other meanings, such as the selection of embryos by sex and other “non-medical” parameters. It can be observed how the scope of reproductive technologies does not find its limit in *reproduction* alone but rather challenges the meanings associated with health, disease, risk, disability, well-being, quality of life, among others (Table 1).

**Table 1:** Changes in terminology and new models of preimplantation genetic testing.

Obsolete terminology	New terminology
PGD = Preimplantation genetic diagnosis	PGT = Preimplantation genetic testing
PGS = Preimplantation genetic screening	PGT-a = Preimplantation genetic testing for aneuploidy
Types of preimplantation genetic testing, as indicated by the physician	
PGT-M = = Preimplantation genetic testing for monogenic conditions	
PGT-SR = Preimplantation genetic testing for structural rearrangements	
niPGT-a = Noninvasive preimplantation genetic testing for aneuploidies	
PGT-P = Preimplantation genetic testing for polygenic risk	

Source: Own elaboration based on The International Glossary on Infertility and Fertility Care.^16^

These changes in how concepts are expressed have concrete practical effects: for example, as they are no longer “diagnoses,” it is necessary to understand how risk assessment will be conducted, and what genetic risk implies in each case. This situation is particularly problematic considering that PGT has expanded to include polygenic conditions, i.e., those multifactorial disorders, often developed during adulthood (such as diabetes, hypercholesterolemia, or mental health conditions), in which the relative weight of genetic and environmental factors as well as the concrete possibility of finding causal genetic variants is debatable. ^17^^,^^18^ This kind of PGT is linked to a growing emphasis on health optimization and extends genetic surveillance to cases in which there is no family history of the disease.^19^ These tests are not exclusively offered to infertile individuals, nor considered as a means to have children any longer. Rather, they are a way to have *healthier children.*^20^ As will be analyzed later, in PGT the risk is often associated with a responsibility. In the most extreme cases, this genetic responsibility of the future parents extends to any characteristic associated with “the best possible life,” which not only would make the use of PGT acceptable but would transform it into a moral imperative, even if the consequence of these actions may exacerbate social inequalities.^19^

Recommendation 3: *Recognize the effects of the language involved in the scientific and clinical discourse associated with reproductive technologies, which are related to the shifts from disease diagnosis to the establishment of a risk, and in the conditions with a clear genetic basis toward the overall optimization of health, with the erasure of environmental factors, especially those of a social nature and related to lifestyles. In this sense, it is possible to frame the growth of PGT within the logic of “healthism”*^21^*and the individual responsibility for health, which, as will be addressed in the following sections, has been subject to criticism from the perspective of collective health.*

## ANALYSIS OF PGT BASED ON THE RISK-SUSCEPTIBILITY MATRIX

As previously mentioned, this shift from “diagnosis” to the analysis of “risk” involves acknowledging how this notion is constructed and understood within this context. It has been shown that, in the context of genetic testing, challenges arise when interpreting results, and, therefore, the way in which information is communicated and conveyed in terms of “risk” is indeed relevant. The discussion of the concept of *risk* often focuses on cognitive biases that hinder the interpretation of percentages or proportions.^22^ In the case of genetic risk estimates, negative reactions and an overestimation of risks (or their interpretation in deterministic terms) are frequently observed.^23^^,^^24^ For these reasons, risk estimators should be communicated in a way that their magnitude is not overestimated by the patient and the potential harmful psychological effects resulting from receiving this information are minimized, so that autonomous and informed decision-making is ensured. 

In this regard, one of the most frequently addressed aspects in the literature on risk communication in the field of health is the use of absolute or relative estimators. To illustrate the difference, we can mention the example of the “*contraceptive pill scare*” that occurred in 1995, when the UK Committee on Safety Medicines announced that the use of contraceptive pills doubled the risk of venous thromboembolism compared to those who did not use them (an increase in the relative risk). However, in absolute terms, this meant an increase of only 1 in 7000 individuals. This incident caused a decline in the use of contraceptive pills, leading to an increase in unintended pregnancies.^25^ Given that relative risks always represent a larger number that can greatly impact public perception and introduce a behavioral bias, it is recommended to always communicate in absolute terms.^26^

However, these recommendations address the way risk is communicated, rather than the problems associated with the concept of risk and the characterization of individuals “at risk.” To begin with, Bianchi^27^ argues that the characterization of “susceptible” or “at risk” individuals focuses on intervention, aiming to lead these individuals toward a “more desirable” future”, and that genetic technologies (among others) represent a “qualitative leap” in the ability to make this future measurable.^27^ In this precise case, as in many others, it involves estimating risks and promoting interventions in the absence of manifest diseases. The concepts of “risk” and “susceptibility” introduce a tension between the individuals and populations that need to be addressed. On the one hand, we can highlight the criticism of the clinical application of risk, which involves this tension between its calculation as an estimator of a population property and its extension to individuals.^28^^,^^29^ Almeida Filho *et al*.^30^ argue against such extension and other assumptions and simplifications in the currently dominant paradigm in epidemiology^30^, which are also present in the assumptions of precision medicine. On the other hand, they problematize the reification of risk and its guilt-inducing use, the reinforcement of “moral and conservative values”, its role in the creation of new markets for the prevention of various risks and the expansion of medical surveillance aimed at extending longevity to the greatest extent possible.^30^ Moreover, along with other authors, they criticize, from a North-South perspective, the categories used, the epistemological assumptions of classical epidemiology, and how the conceptions of health and disease have been “imported” or even imposed in a way that conceals the role of power relationships in health, disease, and care processes, which minimizes the political origins and the consequences arising from the use of these concepts.^30^^,^^31^^,^^32^^,^^33^^,^^34^

Recommendation 4: *Keep a critical perspective on the categories commonly used in epistemology and precision medicine. Latin American bioethics should not only ponder on and discuss issues related to the accessibility, implementation or use of these technologies, nor solely reflect on the communication of results to patients. It should be capable of problematizing the meanings and expectations implied in the various ways of conceptualizing genetic information, in order to contextualize their implications and alternatives.*

## “CENTRAL” BIOETHICAL DILEMMAS ON PREIMPLANTATION GENETIC TESTING

This section describes the bioethical dilemmas that can be found in good practice guidelines and in normative documents of the *central countries* in order to establish a counterpoint when analyzing, in the next section, the translation or *peripheral* issues related to the use and application of these technologies.

The analysis of normative documents from European countries establishes that (with the exception of PGT-P), different PGT variants are usually recommended for individuals who have a *genetic risk*^35^^,^^36^^,^^37^ and wish to prevent the transmission of a pathological phenotype to their offspring, for couples with a high risk of producing embryos with genetic abnormalities, including individuals of advanced maternal age, those experiencing recurrent spontaneous abortions, or undergoing repeated failed *in vitro* fertilizations.

In recent years, the different PGT technologies have sparked various debates and controversies, leading to questions about their potential effects and risks to human health. Among the arguments in favor of PGT, the right to *reproductive autonomy* has been highlighted for those seeking these technologies. Reproductive autonomy is part of the recognized reproductive rights and refers to the individuals’ ability to make informed and autonomous decisions about their own reproduction. Furthermore, its potential benefits include the ability to prevent the transmission of potential genetic disorders, the reduction of pregnancy terminations based on prenatal testing, as well as the potential social benefits of reducing the global burden of severe chronic diseases.^38^

Moreover, the arguments against these technologies question the validity of the benefits of this procedure given its speculative nature, as there is a possibility of error in the results or risk estimates, an inability to predict the long-term development or progress of disease and the potential and still unknown risks arising from the procedures.^39^^,^^40^^,^^41^ There is also concern that the use of this technology for the *selection of embryos* may contribute to the devaluation of certain lives^42^; especially, the lives of those individuals suffering from diseases or carrying specific genetic markers. Thus, it is argued that the problematic notions about the role of genetics as a cause of disease are reinforced, creating a tension between *reproductive autonomy and genetic responsibility*, as mentioned in the previous section. These are long-standing debates. In 1991, Lippman pointed out that prenatal testing technologies, despite being presented as tools for informed decision-making about the health of children, can also exacerbate individual responsibility over the burden of different diseases, reinforcing existing social and economic inequalities. This criticism is also put forward by Latin American authors in the field of collective health.^30^^,^^31^^,^^43^ As also mentioned in the previous section, the trend to attribute genetic foundations to multifactorial conditions encourages the expansion of PGTs and may promote the process of “genetization” of society.^39^

The analysis of the rationales of specific bioethics committees in the global North^35^ recognizes PGT practices as ethically acceptable for those *severe diseases* currently lacking effective treatments.^38^ In particular, the participation of an experienced “genetic counselor”- as defined by the WHO in 1969^44^^)^ - is strongly recommended during the consultation for a proper assessment of potential benefits and risks, which would enable informed and ethically responsible decision-making. The case of PGT-P exhibits specific complexities that make it more controversial, as risks are often reported for treatable conditions, the extrapolation of polygenic risks from one population to another is debatable, and environmental factors are not included in the estimation. For these reasons, along with the pressure exerted on the parents, several authors argue that the difficulties of ensuring reproductive autonomy and informed decision-making are increased in PGT-P^45^^,^^46^ and when considering this type of testing, the same criteria as for other PGTs cannot be applied, although there are currently no specific recommendations.^35^ Nonetheless, this type of tests are being offered by clinics in various countries, including several in Latin America.

Recommendation 5: *Critically reflect on the intrinsic categories in the bioethics field. We should be able to cautiously examine the origin of concepts and debates derived from the field of bioethics and consider how they apply in our contexts. For instance, focus on reproductive autonomy, as the predominant formal procedure of ethics renders invisible the material conditions of the communities in our territories that are not always fulfilled to attain this autonomy. This latter aspect leads us to the following section.*

## BIOETHICAL SUSPICION: NOTHING COMES WITHOUT ITS WORLD

Although rarely made explicit, every bioethical debate and argumentation inherently carries ontological presuppositions. What is asserted, debated, or problematized always has an inevitable starting point in the debaters’ vision; a worldview that, in turn, is shaped by the history of the individuals inhabiting it, their geographical location, institutions, struggles and language. Before adopting a bioethical stance, we must already exist in a tangible and everyday world with its entities, meanings, symbols and objects. The world is, therefore, “the starting category for all other categories.” It is the ordinary, everyday life as a presupposition (what is placed underneath in time: the pre-sub-posed).”^47^ It is what is given, what remains concealed but operates as the practical foundation of our actions and relationships. 

Bioethics is no exception, and therefore, it is crucial to recognize that its discussions and interventions emerge from and are firmly rooted in a specific view of the world. This assertion, that all knowledge, reflection or argument presupposes a specific worldview, compels us to take a step back. It is no longer about evaluating a technology such as PGT from a bioethical perspective, but it is now necessary to cast a critical gaze on the commonly used conceptual frameworks. Thus, it is possible to dialectally achieve a deeper and more meaningful bioethical evaluation of this technology, as we will be able to make a dual judgment: one that analyzes the technology based on the categories offered by the given and unquestioned world; and, another that, by taking a step back, casts *a critical gaze* upon the presuppositions on which the bioethical categories used are grounded.

Recommendation 6: *Review the ontological presuppositions on which the categories and discussions of bioethics are based. Building on the analysis in the previous section, the direction that we propose here for Latin American bioethics is to not lose sight of the fact that the categories employed may not necessarily be extrapolated to other worlds with different realities, such as those of the countries in the global South.*

## WAYS OF AFFIRMING AND REPRODUCING LIFE

Following the same line of thought of the preceding paragraphs, when speaking of different ways of being-in-the-world, we, as Latin American thinkers, begin our reflection from a presupposed ontology. The region has a long history of colonialism and impositions: lifestyles, habits, customs, values, behavior patterns and institutions; ultimately, alien worlds. However, the Latin American reality, the Latin American world, is not and will never be the European or the North American world. There is a symbolic, mythical, historical and psychological background that remains and will remain outside that intended totality or universality, existing as the Other, as the alterity that refuses to be totalized. For this reason, given the multiplicity of worlds that currently coexist in our continent and in the global South, we must be cautious when analyzing bioethics and ask ourselves which particularities of our Latin American world need to be taken into account when evaluating a reproductive technology such as PGT. Or, if formulated as questions, what relevance does the distinction between the global North and global South have when thinking about the appropriateness or desirability of a technology such as PGT from a bioethical perspective? From which worlds do or should the bioethical judgments related to reproductive technologies emerge?

In general, when bioethics reflects on a specific technology, it does so by accepting and taking as a starting point a world governed by a *techno-scientific rationality*, which implies considering, as an *a priori* assumption, that the needs and problems of the communities always find a way out and solutions in scientific knowledge and technological developments.^48^^)^ In some cases, this is regarded as “technological determinism”, when technology is treated as an independent variable that generates ‘effects’ on social processes, or as “social determinism” when it is thought of as a dependent variable where the decisions of the social groups determine the technological change.^49^ In particular, those forms of knowledge, technologies and ways of thinking correspond to the central countries, at which, from a linear and universalistic view, all cultures should arrive.^50^

Therefore, it is natural that when we think about ways of affirming and reproducing human life, the answer is a science-based *technological device or procedure.* It is even assumed that this knowledge and technologies should be universally applied; therefore, there is no difference if we are dealing with knowledge and tools developed in other countries of the world: if it works for a European human being, it equally works for a Latin American human being. Specifically, when referring to the case of genetic testing, it is the lack of data from the populations of non-central countries that has been traditionally problematized. It has even been argued that the greatest ethical and scientific challenges related to the clinical implementation of precision medicine involve the lack of diversity in genomic databases,^51^^)^ which would lead to less accurate risk predictions, but the logic imposed by technology itself is not problematized. Based on this perspective on scientific and technological development, the task ahead will be to analyze the appropriate ways to implement and regulate technology. 

However, as previously suggested, no knowledge or derived technologies can exist in the absence of a world that gives them meaning. Consequently, Latin American bioethics should question the acceptability of presupposed knowledge and developed technology, because they could very well be colonialist mediations, even if they appear as local solutions or as means to “achieve sovereignity” or reverse long-standing inequities between the global North and South. Just as technological universalism has been criticized, so can this criticism be directed at “bioethical universalism”, which, by minimizing the importance of context, eliminates the need to reflect on the uncritical “importation” from the central countries to peripheral ones.^52^ In light of this trend, we believe that, in order to truly address the inequities of Latin American communities, the solution cannot be the uncritical application of technologies, but rather, must involve the consideration of the technologies themselves in the specific context of our societies. Is the technology compatible with the way of being-in-the-world of the Latin American communities? Does it naturally integrate into the life projects of the population? Is it compatible with the various reproduction conceptions held by those inhabiting our territories? When such questions are not posed, bioethics proceeds to discuss and intervene from the positivity of an already presupposed world; a positivity that prevents us from understanding that a culture that does not have access to embryonic manipulation as an ontic possibility for human reproduction cannot judge the ethical aspects arising from the technological application of a device such as PGT. Nor can a culture that lacks guaranteed minimum material aspects that make possible the production and reproduction of its own life and offspring, although the reasons may be different. In either case, the possibilities remain beyond the existential horizon of these communities. 

Recommendation 7: *Reevaluate the ethical foundations that underpin our Latin American community of life and our conceptions of reproduction, so that we can later be able to assess the appropriate mediations that lead us to fulfill our reproductive projects and ultimate goals. This becomes even more important when viewed from the perspective of collective health. We will explain the reasons why below.*

## THE STARTING POINT FOR LATIN AMERICAN BIOETHICAL REFLECTION

The ethical material foundation of a reproductive technology should be the affirmation of life and reproductive health of a community. In this regard, PGT could be considered a mediation that satisfies this foundation. In fact, this is so to a certain extent. However, there is a possibility that such technologies are not the best mediations for the affirmation of life and reproduction within the communities in Latin America - and the argument could also be extended to Africa and specific regions of Asia - for the reasons that we have previously outlined. The everyday reality of the communities in our territories is strongly characterized by exclusion and poverty. In Argentina, for example, official data revealed that poverty reached 39.2% in the last semester of 2022.^53^ If the ethical foundation of this reproductive technology is the affirmation of life and its reproduction, what does this information mean to us as bioethicists who discuss reproductive technologies? Can the context be overlooked and PGT be analyzed as if Latin American populations did not exist in this way? It can be overlooked if a problematic inversion is implemented: placing technological rationality before ethical rationality. One of the potential challenges faced by Latin American bioethics when discussing reproductive technologies from a collective health perspective may precisely be the ontological oversight that leads to an inversion between the foundation and the founded mediation. When analyzing PGT (a mediation for life), we start with the technology (the founded) to discuss its ethical aspects (the foundation) by reversing the order of the reflection. Instead of questioning the meaning of a technology that stems from other worlds, bioethics frequently takes for granted its inclusion and inquire about the ethical ways of implementing it. 

Does this imply that the bioethics of the global South must unconditionally reject all forms of PGT or similar technologies merely because they originated in the global North? No, we do not believe that this should be the case. Instead, it should highlight the importance of taking a complementary approach, one that delves into the underlying ontology and confronts it with the realities of Latin American populations. Only in this way is it possible to think of an ethics from the global South with a collective health perspective, a critical ethics that does not sweep under the rug the fact that a great part of our population does not have nor will ever have access to these technologies. Recovering the ethical foundation that guides us - the affirmation of life and its reproduction - protects us from technological fetishism and opens up the possibility to seek technological mediations that are compatible with our own ontological horizons. And, if a technology emerges in other worlds, remembering this ethical foundation will allow us to take a critical distance when judging the technology in question based on our own facticity. It is indeed a good thing to offer PGT to those individuals with hereditary genetic disorders who live a specific daily reality so that they can affirm their life and reproductive health. However, Latin American bioethics cannot take the European or the North American world as a model to analyze and judge it. Neither should it overlook the issue that a significant part of the communities living in its territories have both their material and symbolic needs denied; for material and symbolic reasons, their life and reproduction are denied. We cannot address this technology if we do not also deal with this issue. 

Recommendation 8: *Start the Latin American bioethical reflections with an analysis of the inequities or inequalities in reproductive health care, which include fragmented health care systems that aggravate the vulnerability of the populations with fewer economic resources, within a scenario characterized by a trend toward the geneticization of the health of certain individuals at the expense of the affirmation of life and reproduction of others, often supported by biomedical discourses within the financial-medical-industrial complex. This critical perspective is our last recommendation for Latin American bioethics when addressing reproductive technologies such as PGT.*

## CONCLUSIONS

As we have attempted to argue throughout this study, we have identified three important ways to problematize the implementation of reproductive technologies such as PGT in the Latin American context. One of them, maybe the most traditional one, questions the biases in knowledge production that have become evident in this case. On the one hand, there is the under-representation of Latin American communities on the databases used for the calculation of genetic risks, and on the other hand, there are difficulties in generating reliable data on the application of these technologies in the countries of the region, considering that the only existing reports are voluntarily disclosed.^10^^,^^54^ As mentioned, the scant available evidence is, to a large extent, produced by the private business sector. Furthermore, it relies on the willingness of the fertility centers to provide data, and the readiness of the various state agents to create registries to control and safeguard the information. These epistemic biases certainly reveal multiple power relationships (private sector-public sector, major urban centers-peripheral regions, global North-global South) and, therefore, collecting data that would enable to implement and regulate these technologies in Latin American populations is presented as desirable or even as an almost “emancipatory” endeavor. 

Secondly, we highlight the need to question the values underlying the advancement of technologies such as PGT. On the one hand, we have mentioned “genetic responsibility” and the notions of “procreative beneficence” that transform technologies of this kind into a moral imperative while creating a mandate as to who can reproduce and how. The expansion of PGT technologies (in terms of the trend to increasingly conduct more preimplantation studies, as well as the advancement of tests such as PGT-P for multifactorial, treatable and adult-onset conditions) reveals this intrusion of “the medical” into everyday life. Among the different types of genetic evaluations, the case of PGT-P may be the most obvious; however, the distinction between medical or non-medical reasons for requesting or conducting preimplantation evaluations is not always clear enough. Other values, such as the desire to conceive genetically-related children, also come into play in the use and regulation of these technologies. 

A third way to problematize these technologies involves reflecting on the technologies themselves and their presuppositions in the Latin American context. This kind of reflection, which is less commonly found in the literature on these topics, is an exercise that can be approached from the perspective of collective health and Latin American bioethics through the critical analysis of health initiatives in their social context.^55^ This leads us to consider not only how to implement these technologies, but also to ask the following questions: Is it desirable to do so? Under what circumstances? What are the presuppositions underpinning these technologies and what logic may they be imposing? Are they an appropriate mediation for the affirmation of life and reproduction of communities? Or in which contexts might they be appropriate?

For each of the aspects mentioned and elaborated here, we have presented recommendations that we hope will be valuable for future research and reflections on Latin American bioethics. What has been addressed in this article does certainly not exhaust the possibilities or instances related to what can and should be discussed from the global South when analyzing a reproductive technology. We are aware that precision medicine is rapidly expanding and has already knocked on the door of Latin America. We hope that this contribution will help ensure that nobody dictates or imposes an answer to that call.
